# The changing phenotype of microglia from homeostasis to disease

**DOI:** 10.1186/2047-9158-1-9

**Published:** 2012-04-24

**Authors:** Xiao-Guang Luo, Sheng-Di Chen

**Affiliations:** 1 Department of Neurology, First Affiliated Hospital of China Medical University, Shenyang, 110001, China; 2 Department of Neurology & Institute of Neurology, Ruijin Hospital affiliated to Shanghai Jiao Tong University, Shanghai, 200025, China

**Keywords:** Microglia, Neuroprotection, Phenotypes, Senescence, Crosstalk

## Abstract

It has been nearly a century since the early description of microglia by Rio-Hortega; since then many more biological and pathological features of microglia have been recognized. Today, microglia are generally considered to be beneficial to homeostasis at the resting state through their abilities to survey the environment and phagocytose debris. However, when activated microglia assume diverse phenotypes ranging from fully inflamed, which involves the release of many pro-inflammatory cytokines, to alternatively activated, releasing anti-inflammatory cytokines or neurotrophins, the consequences to neurons can range from detrimental to supportive. Due to the different experimental sets and conditions, contradictory results have been obtained regarding the controversial question of whether microglia are “good” or “bad.” While it is well understood that the dual roles of activated microglia depend on specific situations, the underlying mechanisms have remained largely unclear, and the interpretation of certain findings related to diverse microglial phenotypes continues to be problematic. In this review we discuss the functions of microglia in neuronal survival and neurogenesis, the crosstalk between microglia and surrounding cells, and the potential factors that could influence the eventual manifestation of microglia.

## Table of contents

I. Introduction

II. The origin of microglia

III. Microlgia the dual natures of neurotoxicity and neuroprotection

IV. Crosstalk between microglia and other brain cells

1. Cross talk between microglia and neurons:neurons as regulators of microglial activation

2. Cross talk between astrocytes and microglia: reciprocal influences

3. Microglia-T cell crosstalk:key determinants for the trend of immune response

V. Whether microglial activation is neurotrophic or neurotoxic is context-dependent

1. Aging can result in microglial dysfunction and subsequent neurotoxicity

2. The timing of activation is an indispensable determinant of microglial function

3. Activation states of microglia

4. The stimulus type is another turning point for microglial function

VI. Microglia and neurogenesis

VII. Conclusion

## Introduction

Microglia are generally considered the immune cells of the central nervous system (CNS) and account for 10% of the total glial cell population in the brain. In a normal physiological environment, they work as sentinel cells by continually screening the brain tissue; they actively participate in pathological processes by changing morphology, expressing various antigens and becoming phagocytic. During the past 20 years, thousands of papers have been published describing both the detrimental and beneficial roles of microglia in various brain disorders, from acute infection or stroke to the long and chronic process of neurodegeneration. Microglia have been firmly established as a key cellular component involved in the eventual outcome of inflammation and eventually contribute to the chronic neurodegeneration; The physiology and signaling of microglia have been comprehensively reviewed by Kettenmann’s series papers[1-6], however, the regulation of microglial activity is a highly complex system, and the responses of microglia are tailored in a multi-factor dependent manner, and which are the focus we try to review in this paper.

## The origin of microglia

The precise origin and cell lineage of microglia has been a long time debate. So far two most important hypotheses for microglial origin have been held: “neuroectodermal” or “myeloid-monocytic”. Even though the latter has been more widely accepted now, the neuroectodermal hypothesis remains interesting. Skoff [7] detected “multipotential glia cell” with a rat model of optic nerve degeneration and optic nerve development, these cells were demonstrated to originate from neuroectodermal matrix cells, and Kitamura later confirmed this result by describing a continuous morphological transition between glioblasts and ramified microglia in the developing gray matter of hippocampus [8]. The hematopoietic origin of microglia also received a lot attention, the presence of bone marrow Mac-1 positive cells were demonstrated in the brain of embryonic and adult mice, and these cells were proved to be the progenitors for microglial cells [9], also transplantation of GFP + mice bone marrow cells in GFP- host mice revealed the presence of many GFP + microglia throughout developing and/or inflamed CNS [10,11], which strongly suggest the hematopoietic stem cells as one of the origins for replenishment of microglia in the neuropathology. Additionally due to the high similarity in marker expression and phagocytosis behavior between circulating monocytes and microglia, people speculate the monocytic origin of microglia, and a couple of experiments have been performed to show the appearance of labeled monocytes in the developing [12] or inflamed brain [13]. In many cases, the peripheral macrophages are considered to be the orthologue [14,15] or backup of microglia and infiltrate the brain to supplement microglia, thus to some extent peripheral macrophages mirror the behavior of microglia in the brain and Monocyte-derived Macrophages (MDMs) from patients have been used as a substitute of microglia in many studies [16-18].

## Microglia: the dual natures of neurotoxicity and neuroprotection

Neuroinflammation has long been considered a mediator of secondary damage following a small injury to the CNS. As the primary immune cells in the brain, microglia are expected to take active roles in the damage process. The presence of activated microglia within injured brain regions and in post-mortem tissue from patients having various neurodegenerative disorders has led to the assumption that all reactive microglia contribute to an adverse and degenerative process. Further studies describe destructive roles for microglia by demonstrating the release of a range of neurotoxins from microglia that includes pro-inflammatory cytokines [19-21], nitric oxide [22,23] and reactive oxygen species [24,25]; the inhibition of microglial activation in various experiments results in the attenuation of neurotoxic events and improves neuronal survival. In various neurodegenerative disorders, the over-activation of microglia is considered to be a key causative factor in the process or, at a minimum, to promote the neuropathology. For example, in Alzheimer’s disease, microglia activated by amyloid-β(Aβ) protein, the hallmark of the disease, release neurotoxins and potentiate neuronal damage, and this microglial over-activation is an early event that precedes neuropil destruction [26]. The activated microglia cluster around or penetrate the neuritic plaques [27], supporting a critical role of microglial activation in the pathogenesis and progression of the disease. In Parkinson’s disease (PD), an increased number of activated microglia are present in the vicinity of degenerating neurons [28] in the substantia nigra [29], which is particularly deleterious to dopaminergic neurons due to their glutathione deficiency [30]. A single injection of lipopolysaccharide (LPS) to activate microglia in the substantia nigra region led to a progressive, preferential and irreversible loss of dopaminergic neurons [31-33], even though LPS itself did no direct harm to the neurons, indicating that the over-activation of microglia is capable of inducing neuronal death in the absence of other pathological stimulation. All of the evidence described above supports the hypotheses of the neurotoxic features of microglia.

However, as the sentinel and essential cells of the CNS, it is unlikely that microglia would function to damage neurons in all scenarios. Once stimulated the microglia migrate rapidly to the injury site along the chemokine gradients in vitro [34] and also in response to chemoattractants including ATP and NO released directly or indirectly by the injury [35] to exert effect on the survival of neurons. In fact, some specifically designed experiments have begun to uncover the neuroprotective roles of microglia, and more studies are emerging to show beneficial functions of microglia. Firstly, studies have demonstrated instructive roles for microglia in the developing brain for neuronal differentiation [36,37] and in the regulation of neuronal apoptosis [38] through the production of neurotrophins [39]. Secondly, in the adult brain, resting microglia, which are characterized by many fine perpendicular processes extending from a few long prolongations, have been regarded as sensor cells for the detection of abnormalities or changes in the brain [40] and help to maintain environmental homeostasis. Lastly but most importantly, activated microglia have also been shown to perform neurotrophic functions following neuronal injury. One compelling study supporting this finding involves the axotomy of peripheral nerves (facial or optic), where a rapid microglial response is exhibited with the efficient clearance of myelin debris that contained inhibitory molecules of axon growth, finally leading to successful axonal regeneration [41]; the inhibition of this microglial response to facial nerve axotomy impairs neuronal survival [42]. In addition, in neonatal mice administered MPTP, highly activated microglia show neurotrophic potential towards dopamine neurons [43] and after traumatic injury, clear glutamate without evoking inflammatory mediators [44]. The benefits of microglial activation are further demonstrated by the exacerbation of neuropathology in inducible mouse models that are deficient in microglia [45,46], the finding of protective microglia in cases of cerebral ischemia [47] and multiple sclerosis [48] and the fact that transplantation of microglia can help to enhance neurite growth and functional recovery after CNS injury [49,50]. The bunch of factors that can activate microglia and the differential behavior of microglia in various conditions have been listed in Table 1 &2. The above studies clearly demonstrate that microglia can be neurotrophic in the proper situations; there might be a third possibility that microglia are activated by simply reacting to pathogenic stimulation and takes very limited roles in the neurological disorders, in such case the activation of microglia is solely a result of pathogenic stimulation and work as a by-stander that either involved passively during the whole process or even go to apoptosis by some other signals. Thus These activated microglia might have different phenotypes. However, the details of what conditions induce microglia to take beneficial phenotypes remain unknown. Many factors are likely involved in determining the eventual outcome of the manifestation of microglia, including their interaction with neurons or astrocytes in the same environment, age-related dysfunction of microglia, activation timing, and the activation state of the microglia, which we will be discussing below.

**Table 1:** Factors that can activate microglia

Substance that can activate microglia	Reference
**Pathological conditions**	
hypoxia	Morigiwa et al., 2000 [51]
tumor	Bosco et al., 2011[52]
Ischemic insult	Hur et al., 2010 [53]
Nerve injury	Maeda et al., 2010 [54]
**Proteins**	
α-synuclein	Lee et al., 2010; Su et al., 2008; Zhang et al., 2005 [55-57]
amyloid-beta	Jana et al., 2008 [58]
fibrinogen	Piers et al., 2011 [59]
Thrombin	Lee et al., 2005 [60]
Tissue plasminogen activator	Siao et al., 2002 [61]
Matrix protein (vitronectin, fibronectin, MMP-3)	Milner et al., 2007; del Zoppo et al., 2007; Kim et al., 2005 [62-64]
**Chemicals**	
Adenosine Triphosphate	Matsui et al., 2011 [65]
Toxins (MPTP, Rotenone, Paraquat)	Yasuda et al., 2008; Gao et al., 2002; Wu et al., 2005 [66-68]
Alchohol	McClain et al., 2011 [69]
Dopamine quinone	Kuhn et al., 2006 [70]
Berberine	Lu et al., 2010 [71]
lipopolysacchride	Jung et al., 2010; Meng et al., 2008; Xu et al., 2009 [72-74]
**Cytokines**	
TNF-α	Iribarren et al., 2005 [75]
IL-6	Krady et al., 2008 [76]
IL-12	Tamakawa et al., 2004 [77]
IL-3	Natarajan et al., 2004 [78]
IFN-Υ	Rozenfeld et al., 2005; Hall et al., 1999 [79,80]
**Others**	
gangliosides	Kim et al., 2009; Min et al., 2004 [81,82]
Kalic acid	Zheng et al., 2010; Zhu et al., 2010 [83,84]

**Table 2:** Behavior of microglia in different conditions

Conditions	Microglia function
**In steady state**	
Healthy resting state	Surveillance, homeostasis [85] Fixed cell and motile processes, minimal expression of cell surface markers and release of cytokines and chemokines, not involved in Phagocytosis
**In disease state**	
*Neuroprotective*	
Axotomy of the optic nerve	Efficient clearance of myelin debris [41]
Traumatic injury	Clear glutamate without evoking inflammatory mediators [44]
Ischemia	Synthesis of tumor necrosis factor, engulfment of harmful invading neutrophil granulocytes [86]
Alzheimer’s Disease	Internalize and degrade amyloid beta [87]
Multiple sclerosis	Secrete soluble mediators that trigger neural repair and usually contribute to the creation of an environment conductive for regeneration [48]
*Neurotoxic*	
Parkinson’s disease	Releasing various kinds of noxious cytokines, reactive oxygen species [88]
Multiple sclerosis	Express iNOS [89] and generate toxic ROS which might injure neurons
Alzheimer’s disease	Produce of chemokines, neurotoxic cytokines and reactive oxygen an dnitrogen species that are deletrious to the CNS [90]

## Crosstalk between microglia and other brain cells

Microglia have been considered to be the first line of defense in the CNS [91], a hypothesis that has been supported by the finding that microglia actively screen their microenvironment with highly motile processes; thus, the brain is under continual surveillance by microglia. To do this with high efficiency, microglia must be variable, adaptive to their environment and capable of integrating various inputs and responding appropriately [92,93]. All of these processes require significant interactions with other components within the same environment, including neurons and astrocytes.

### Crosstalk between microglia and neurons: neurons as regulators of microglial activation

When we talk about whether microglia are neuroprotective or neurotoxic, we only refer to the influence of microglia on neurons. However, many studies indicate that neurons are not merely passive targets of microglia but rather exert control over microglial activities [94]. There are considerable interactions between neurons and microglia. For example, Polazzi hypothesized that activation of microglia as a consequence of neuronal injury is primarily aimed at neuroprotection, with the loss of specific communications between neurons and microglia leading to the neurotoxic behavior of microglia [95]. Accumulating evidence demonstrates that there is significant information exchange between neurons and microglia. Depending on whether they are healthy or injured, neurons send “on” or “off” signals to influence microglial activation. On one hand, the activation of microglia by neuronal injury or degeneration has been widely reported [91,96]. On the other hand, in the healthy brain, microglial activation is tightly restricted by signaling from neurons. CD200-CD200R has been identified as one of the critical pathways in attenuating microglial activation. CD200 is a member of the immunoglobulin superfamily and is expressed on the neuronal membrane surface, while the CD200 receptor (CD200R) is primarily present in the macrophage lineage, which includes microglia [97]. The disruption of CD200-CD200R interactions results in an accelerated microglial response, whereas intensified CD200-CD200R interactions contribute to an attenuation in neurodegeneration [98]. In mice that have had CD200 selectively removed from neurons, microglia exhibited an activated phenotype and were numerous upon facial nerve transaction; damaged CD200-deficient neurons elicited an accelerated microglial response, which demonstrated a loss of the neuronal inhibitory signal for microglial response [97]. Apart from direct interactions through receptor-ligand combinations, electrical activity and soluble factors released from intact neurons also maintain microglial quiescence. In a neuron-glia co-culture, the blockade of neuronal electrical activity by tetrodotoxin or a glutamate receptor antagonist facilitated microglial activation induced by IFN-γ [99]. Soluble molecules from neurons such as neurotrophins and anti-inflammatory agents down-regulate antigen expression on cultured rat microglia [99,100]. Additionally, released factors from neurons can also influence the survival of microglia. Fukui et al. demonstrated that treatment with conditioned media from mature neurons significantly induced the death of microglial cells independent of LPS, while heated neuron-conditioned media or low-calcium-ion media prevented the death of microglia [101], indicating that specific factors released from neurons exert detrimental effects on microglia. It has been demonstrated that microglial cells undergo apoptosis following peripheral nerve injury [102-104] or in cases of experimental autoimmune encephalomyelitis(EAE) [105]Injured neurons induced either neuroprotective or neurotoxic behaviors in microglia depending on the manner of injury [91,106-109], providing strong evidence to support the hypothesis of crosstalk between neurons and microglia. Thus, microglia are not merely surveyors of brain tissue but also receive and actively respond to signals from neurons.

### Crosstalk between astrocytes and microglia: reciprocal influences

Although less obvious than the crosstalk with neurons, the interactions between microglia and astrocytes are far from simple and are also crucial for our understanding of how microglia respond to their environment and exert influence on neuronal degeneration or regeneration. Several studies have demonstrated the substantial influence of astrocytes on microglial activation [110]. The induction of microglia by Trimethyltin or Borna disease virus-infected neurons is dependent on the presence of astrocytes [111,112]. Astrocytes play neuroprotective roles by modulating microglial cell activity and decreasing their cytotoxicity [113,114]. The expression of IL-12 and the production of inducible nitric oxide synthase (iNOS) in activated microglia have been reported to be suppressed by astrocytes or conditioned media from astrocytes [82,111,115-117], delineating the signals from astrocytes that affect the activities of microglia. Furthermore, the communication between these two types of cells is two-way; microglia both receive and give signals, as pro-inflammatory cytokines released from microglia inhibit gap junctions and down-regulate connexin 43 expression in astrocytes [118-120], which enhances astrocyte survival. In another study, comparative proteome analysis was performed on astrocytes that were treated with conditioned media from quiescent or activated microglia. Following culture in activated-microglial media, the anti-oxidative enzymes expressed in astrocytes were up-regulated, and these astrocytes were protected against oxidative stress. This result gave insight into the complex intercellular events that take place during neurological disorders [121]. As in many pathological conditions in the central nervous system such as in neurodegeneration [122], microglia, activated earlier than astrocytes, promote astrocytic activation through IL-1which is mostly from microglia [123]. On the other hand, activated astrocytes not only facilitate activation of distant microglia via calcium wave [124,125], but also inhibit microglial activities [126]. Additionally, it was observed that activated-microglial-conditioned media increased astroglial proliferation [127], down-regulated the astroglial metabotropic glutamate receptor [128] and induced astroglial brain-derived neurotrophic factor (BDNF) and IL-6 gene expression [129]. Taken together, the importance of microglial activities lies in that they not only exert direct effects on neuronal survival, but they also affect the responses of other supporting cells in the same environment.

### Microglia-T cell crosstalk: key determinants for the trend of immune response

The entire immune response consists of the cooperation of the innate and adaptive immune systems. In the brain, it has been postulated that the beneficial or destructive outcome of the local microglial (innate) response is determined by a well-controlled dialogue between the innate and the adaptive immune players, which are, in most cases, the microglia and T cells. Activated T cells can cross the blood–brain barrier and interact with resident microglia in the parenchyma [130]; these microglia have been characterized as myeloid progenitor cells that can differentiate into macrophage-like or dendritic-like cells [131] and thus work crucially as the principal APCs [85] in the CNS. Monsonego et al. demonstrated that IFN-γ-treated microglia serve as efficient Aβ antigen-presenting cells (APCs) of both Aβ1-40 and Aβ1-42, mediating CD86-dependent proliferation of Aβ-reactive T cells [132]. The activated T cells then exert effects in the injured neural tissues by altering the reactive microglial phenotypes and inducing the astrocytic expression of growth factors or modulating microglia to act as glutamate scavengers [44] to improve neuronal survival [133,134]. In a model for optic nerve injury, the passive transfer of regulatory CD4 + CD25+ T cells was either destructive or beneficial depending on the genetic background of the mice tested, which determines the differential interaction of T cells with microglia and thus the different T cell-mediated microglial phenotypes [133]. Kipnis even observed that both the suppressor and the effector activities of T cells could be mediated through dialogue with microglia in the condition of neurodegneration [135], The entire scenario of crosstalk between T cells and microglia could be described as the following: microglia are initially activated by pathological stimuli during acute or chronic injury to the brain; if the activation occurs with the proper timing and mode and is well-controlled, the activated microglia will work as APCs [133] to stimulate Treg cells that eventually modulate the microglial activation directly or indirectly and affect the milieu balance between neurotrophism and cytotoxicity [44,136,137].

## Whether microglial activation is neurotrophic or neurotoxic is context-dependent

After considerable time and research, we have recognized the “double-edged sword” nature of microglial cells. On one hand, significant evidence from in vitro and *in vivo* studies has associated neuronal injury with microglial activation [138-141]. This evidence results from an inflammatory phenotype of microglia releasing neurotoxic factors, mediators and reactive oxygen species [138-141]. On the other hand, several other studies have highlighted the beneficial and important roles of microglia in neuronal regeneration, repair and neurogenesis [142-146]. These seemingly paradoxical results cannot be directly compared, because they come from different experimental sets that vary in terms of the stimulus, timing of microglial activation and age of animals. Thus, whether microglia have positive or negative effects on neuronal survival is context-dependent.

### Aging can result in microglial dysfunction and subsequent neurotoxicity

**Figure 1:**
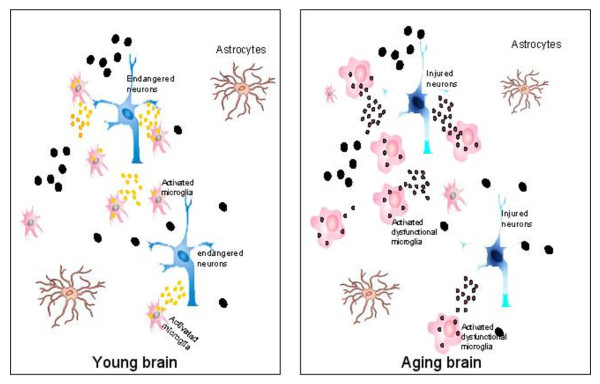
**Age-primed microglia hypothesis of Parkinson’s disease.** Microglia functions differentially in the young (left) and aged (right) brain. Left: when facing pathogenic stimuli (large black dots), the healthy microglia in the young brain respond by releasing neurotrophic factors (small yellow dots) to support the endangered dopaminergic neurons and limit neuronal damages. Right: in the aged brain oxidative stress and inflammatory factors (small black dots), which damage the vulnerable dopaminergic neurons and eventually lead to neurodegeneration. (From Luo et al.,2010 with permission).

There are studies suggesting that senescence in microglia causes them to function abnormally and that the destructive roles of activated microglia in the aged neurodegenerative brain may result from age-associated microglia senescence, causing a failure of the aged microglia to respond correctly to stimuli [147,148] and eventually promoting neurodegeneration [149] (Figure 1). The most prominent and also the initially identified feature of microglial senescence is the morphological alteration described as “dystrophy” [150]. Characteristics of “dystrophic” microglia observed in the aged brain include de-ramification (the loss of finely branched cytoplasmic processes), cytoplasmic beading/spheroid formation, shortened and twisted cytoplasmic processes, and instances of partial or complete cytoplasmic fragmentation [150]. Such dystrophic microglia were prevalent and extensively distributed in the brain of older human subjects [150,151], whereas normally ramified microglial morphology with only rare instances of dystrophic microglia is observed in the young brain [148]. These observations provide initial evidence for the age-associated changes in microglia in the healthy elderly brain. Telomere shortening, a marker of aging, has also been demonstrated in microglia in the aged brain in Flanary’s study, who reported that microglial cells in rats exhibit significant telomere shortening and a reduction in telomerase activity during normal aging [152]. More importantly, microglial senescence is also manifested by functional alterations, such as an altered inflammatory profile, increased immuno-phenotypic expression, and the switch from neuroprotective in the young brain to neurotoxic in the aged brain upon activation [147]. Also, the timing of microglial proliferation and presentation in the injured aged brain is distinct from that in the young brain. For example, Conde et al. reported that microglial proliferation rates in the aged rat brain were significantly higher than in the young rat brain four days after axotomy of the facial nerve [148]. The distinct pattern of the microglial response to injury in the aged brain has also been recorded in the 1-methyl-4-phenyl-1,2,3,6-tetrahydropyridine (MPTP)-induced model of neurotoxicity [153], the model of controlled cortical impact (CCI) [154], cortical stab injury [155]and transient retinal ischemia [156]. Although more attention has been paid to the dysfunction of aged microglia, many critical questions remain unanswered. Some of these questions are: whether the activated state of microglia in the aging brain is concurrent with or secondary to microglial dystrophy; which specific function of microglia is primarily affected by microglial dystrophy, how it is affected and what is the direct consequence of the affected function; and whether the deterioration of a specific microglial function is more related to neurodegeneration than others. Clearly, more research is needed to answer these questions.

### The timing of activation is an indispensible determinant of microglial function

Another important element that critically determines the destructive or neuroprotective role of microglia is the timing of their activation. Because large and very complicated communications pathways exist between immuno-competent cells and cytokines in the CNS, the timing of microglial activation leads to diverse trends and outcomes related to the entire inflammation event. In a model of optic nerve crush injury, Shaked et al. found that an earlier onset of phagocytic activity and antigen presentation by microglia results in a resistance to injury and neurons survived [133]; the early, moderate, transient and well-controlled activation of local microglia caused them to function as APCs, leading to the communication with Treg cells that subsequently proves to be neuroprotective through the modulation of microglial activation states [133]. In a multiple sclerosis (MS) model of experimental allergic encephalomyelitis (EAE) [157], the inhibition of microglial activation through tPA knockout (tissue plasminogen activator, an essential element for microglia activation) leads to a delayed onset of the disease but increased severity and delayed recovery from the neurological dysfunction, which suggests that microglial activation is harmful during the onset of the disease but beneficial in the recovery phase [157]. Furthermore, when microglial activation was either stimulated or inhibited at different stages, the disease progression was attenuated or exacerbated accordingly [158]. For example, the inhibition of microglial activation at EAE onset, rather than prior to EAE induction, markedly decreased EAE progression, while the stimulation of microglial activation prior to the onset of EAE promotes lower-level EAE and an earlier recovery from symptoms. Together, these findings suggest different roles for microglial activation during various phases of the disease and that different timing of microglial activation dramatically affects whether microglia will be neuroprotective or deleterious [158]. Similarly, in an oxygen-glucose deprivation model, the time window of microglial neuroprotection has been estimated to up to 48 hour after injury, while the pre-stimulation of microglia with LPS before the injury fails to induce microglial-mediated neuroprotection [86]. It has been proposed that the effects of the early activation of microglia on disease progression could be beneficial through phagocytic activity and antigen presentation, recruitment and interactions with the adaptive immune response and the induction of protective autoimmunity [133]. Furthermore, the balance between protective autoimmunity and autoimmune disease may be determined by the timing and intensity of microglial activation [133]. As the immuno-competent cells in the CNS, microglia are critical determinants of the outcome of injury, and the timing of microglial activation appears to be crucial to the outcome of the injury. Thus, any interference with microglial activation in an attempt to affect the disease course clearly must be temporally-restricted.

### Activation states of microglia

Two distinct phenotypes of macrophages have long been known to play different roles in the inflammatory context. Classically-activated macrophages, characterized by the involvement of T Helper type 1 (Th-1) cytokines such as interferon-γ, promote the release of various pro-inflammatory cytokines and thus exacerbate the inflammation. Alternatively, activated macrophages predominate in the T Helper type 2 (Th-2) microenvironment and tend to soothe the inflammation. Thus, the behavior of macrophages is dictated by their phenotype, which may eventually affect the beneficial or detrimental roles of macrophages during inflammation. Similarly, research over the past few years has established that microglia do not constitute a single, uniform cell population, but rather comprise a family of cells with diverse phenotypes; some are neuroprotective while others are destructive [92]. So far, three distinct functions have been proposed for microglia. The first is the classical activation state of microglia, which, accompanied by the induction of receptors that participate in the innate immune response [159], is responsible for the pro-inflammatory milieu, and has been linked to neurotoxic effects in the brain. The second is alternatively activated microglia, which are associated with the production of anti-inflammatory cytokines in the resolution phase of the inflammatory response. Recently, the third activation state of microglia has been identified: it overlaps with and is complementary to the alternative activation and is called acquired deactivation [160,161]. This is another activation state that promotes immunosuppression and is associated with the anti-inflammatory and functional repair phenotype .Both alternative activation and acquired deactivation down-regulate innate immune responses and have similar gene profiles; the most prominent difference is that acquired deactivation is induced by the exposure of microglia to apoptotic cells or to TGF-β or IL-10, while IL-4 and IL-13 induce alternative activation [160,161]. It has been observed that multiple activation states of microglia coexist in certain chronic inflammations due to parasitic disease [162], in which the balance between classical activation and alternative activation/acquired deactivation states is of “benefit” to both host and parasite: the host benefits from reduced self-damage, and the parasite eventually survives within the host. Neurodegenerative disorders are also associated with chronic inflammation and the coexistence of various activation states. For example, in AD, some levels of classical activation may be required to limit the brain levels of Aβ despite the risk of self-damage [163], while alternative activation of microglia in AD may foster the protection of the surrounding tissue from immune damage even though it may facilitate Aβ deposits. Similar studies [164-166] have shown that the immune cells in the vicinity of amyloid deposits in AD express mRNA and proteins for pro-inflammatory cytokines, leading to the hypothesis that microglia demonstrate classical activation in AD, while Colton et al. found increased mRNA expression of alternative activation-associated gene profiles in microglia in both the AD brain and an AD mouse model [167], suggesting the presence of multiple activation states of microglia during neurodegeneration. However, the recognition of heterogeneous phenotypes of microglia only raises more questions: what instructs microglia to acquire a particular phenotype; can any conversion occur between these phenotypes; and is it possible to avoid or at least change the commitment to a destructive phenotype? All of these questions are difficult to answer with our current knowledge of microglia; more extensive work is warranted before we can reach a conclusion.

### The stimulus type is another turning point for microglial function

As an active sensor in the brain, microglia respond to even minor stimuli; however, different types of stimulation may also lead to different actions of microglia and thus be either harmful or beneficial to neuronal survival. In a neonatal mouse MPTP-induced brain injury model, microglia activated by systemic administration of LPS were shown to be neuroprotective. In contrast to the MPTP model, LPS-activated microglia in neonatal mice receiving a stereotaxic injection of ethanol into the striatum were shown to be neurotoxic, and systemic LPS administration in the ethanol-injury model caused a marked increase both in the volume of necrotic lesions and in the number of degenerating neurons in the striatum [168]. Even with the same stimuli, the degree can also determine microglial release of toxic versus protective effectors [169]; neurotoxic cytokines and ROS were released from microglia only in response to mild neuronal injuries, while trophic microglial effectors such as BDNF and GDNF were up-regulated in response to all degrees of neuronal injury [169]. Additionally, different types of pain resulted in differing activations of microglia [170].

So far, what we know is that not all microglia respond in the same way, even to the same stimulus, and microglial function is tailored in a context-specific manner [171]. Numerous elements are involved in this context; most likely there are many more beyond what we have discussed here. Identifying these elements and clarifying their interactions or crosstalk with microglia is essential before we are able to design a strategy to control inflammation through the manipulation of microglia. The simple therapy of inhibiting all microglia without differentiating their function in a context-dependent manner surely should be abandoned.

## Microglia and neurogenesis

It has been long recognized that the birth of new neurons within the postnatal brain continues throughout life and remains as a potential source of replacement cells in the CNS for the treatment of disease. The microenvironment or the niche in which neural progenitor cells live critically influences the process of neurogenesis, which spans several steps including the proliferation of stem or progenitor cells; the survival of immature or mature neurons; the migration of new neuroblasts to their appropriate locations; and the differentiation of neuroblasts to a neuronal phenotype and the construction of synaptic connectivity [172]. As an important component of the brain microenvironment and due to their invariant participation in most pathological processes in the CNS, microglia are increasingly implicated as a potential non-neural regulator of neurogenesis, as demonstrated by circumstantial evidence [144,172]. However, just as in the debate over the neuroprotective or neurotoxic nature of microglial activation, whether microglia support or damage the survival and development of neural progenitor cells also remains controversial. On one hand, microglia were shown to play instructive roles during postnatal neurogenesis in the neurogenic niche either by influencing the differentiation of stem cells toward a neuronal phenotype or by directing their migration [144,173-175]. On the other hand, multiple studies have demonstrated the deleterious effect of microglial activation on neurogenesis [176,177] and the effective restoration of neurogenesis though the blockade of microglial activation.

In the two situations of neurogenesis and neuronal survival, similar factors are shared, leading microglia to take supportive or detrimental roles. Among these factors, the most prominent is the microglial activation phenotype that is associated with different cytokine profiles. When acutely activated by either LPS or injury, microglia that release the pro-inflammatory cytokines IL-6, TNF-α or IL-1β usually down-regulate the differentiation or proliferation of neural stem cells or induce the aberrant migration of newborn neurons [178]. This group of inflammatory cytokines has been proven to inhibit neurogenesis [176,177,179]; conversely, blocking antibodies to these pro-inflammatory cytokines (such as IL-6 [177]) or the use of monocycline to mitigate the microglial activation simply restores neurogenesis [176]. In contrast, microglia that are activated by anti-inflammatory cytokines such as IL-4 or TGF-β increase neurogenesis in vitro or the differentiation of neural stem cells (NSCs) *in vivo*[180,181]. Neurotrophins, such as IGF-1, were identified [181] in anti-inflammatory cytokine-activated microglia and were proposed to be one of the mechanisms underlying this pro-neurogenic activity of microglia [182,183]. However, just like the dual roles in neuroprotection, whether a specific cytokine-activated microglial cell will take a pro- or anti-neurogenic role is also context-dependent. For example, microglial cells activated by IFN-γ, a pro-inflammatory cytokine can be neurotoxic or supportive of neurogenesis, depending on the concentration of IFN-γ [184]. TGF-β, which is considered to be beneficial to neurogenesis, can actually exert a negative influence on neurogenesis when it is chronically produced in the aged brain [185]. Additionally, if other cytokines exist in the same niche simultaneously, the outcome will be determined by the balance among the various cytokines; some authors have concluded that activated microglia are not pro- or anti-neurogenic *per se*, but the balance between pro- and anti-inflammatory secreted molecules influences the final effect of microglial activation [172,180]. However, in which situations the microglia will release pro- or anti-inflammatory cytokines is complicated and is affected by multiple factors such as the injury type, the phase of disease or inflammation, and crosstalk with other regulating components, including neural precursors; this is similar to the question of whether microglia will be neuroprotective or neurotoxic. Most likely the same inflammatory scenario that induces neurodegeneration would also inhibit neurogenesis, while a situation that favors neuronal survival would also support neurogenesis. Interestingly, even in a high-inflammation environment, such as two days after a Trimethyltin-induced acute injury in the hippocampus, significant neurogenesis can be detected [186,187], suggesting a complicated system of neurogenesis regulation beyond the inflammation scenario.

Cumulative studies have found an age-related decline in neurogenesis, both in the aged adult and in the diseased brain. Because aging may contribute to microglial dysfunction and neurotoxicity, as we discussed previously in this review, one could assume that microglial dysfunction may also be involved in the downregulation of neurogenesis in the aged or diseased brain [188,189]. Even though very few studies have focused on the effect of microglial dysfunction on neurogenesis, we can still find a clue from Zhu’s study that the difference in microglia function patterns between the immature and juvenile brain might be related to a decrease in neurogenesis in the juvenile brain [190]; however, stronger evidence from the direct comparison of microglia-associated neurogenesis between aged and young brains is needed to support this view.

Another important element regulating the activities of microglia is the T cell, which comes from the peripheral adaptive immune system and enters the CNS by extravasating across the endothelium of the choroid plexus into the cerebrospinal fluid [191]. The interaction of T cells with microglia in the injured spinal cord correlates with enhanced neuronal survival [184], and rapidly recruited T cells in the middle cerebral artery obstruction (MCAO) model increased hippocampal and cortical neurogenesis by modulating the microglial response and through the production of IGF in the sub-acute phase [192]. Hippocampal neurogenesis was associated with the recruitment of T cells and microglial activation. Immune-deficient mice show impaired neurogenesis in the hippocampus, but this deficiency was attenuated and neurogenesis boosted by T cells recognizing a specific CNS antigen [193]. The cellular source of IFN-γ and IL-4 *in vivo* is likely to be T cells, therefore it is reasonable to assume that the T cell-mediated immune response is an integral part of the regulation of microglial phenotype or function, and thus can influence neuronal survival or neurogenesis directly or indirectly.

## Conclusion

From an increasing number of studies of diverse microglial activity in different experimental sets, we are beginning to appreciate the heterogeneity of microglial functions that have either beneficial or detrimental roles in specific physiological or pathological environments. Whether microglia are committed to one function from the very beginning or if there is any conversion between different phenotypes remains elusive and the factors that initiate this commitment or promote its conversion are far from being clarified. Due to the invariant critical participation of microglia in most diseases, ongoing research to uncover these questions is warranted; before we are sure about the answer, any potential strategies targeting microglia to manipulate inflammation and modify a disease course are unrealistic.

## Competing interests

The authors declare that they have no competing interests.

## Authors’ contributions

XL drafted the manuscript, SC critically revised the manuscript. All authors read and approved the final manuscript.
